# Assessing Different Histological Preparations for Reconstruction of Astrocyte Tridimensional Structure

**DOI:** 10.3390/cells13110969

**Published:** 2024-06-03

**Authors:** Sara Barsanti, João Filipe Viana, Alexandra Veiga, João Luís Machado, Daniela Sofia Abreu, José Duarte Dias, Susana Monteiro, Nuno A. Silva, Luísa Pinto, João Filipe Oliveira

**Affiliations:** 1 Life and Health Sciences Research Institute (ICVS), School of Medicine, University of Minho, 4710-057 Braga, Portugal; id10418@alunos.uminho.pt (S.B.); id9532@alunos.uminho.pt (J.F.V.); pg49202@alunos.uminho.pt (A.V.); id10766@alunos.uminho.pt (J.L.M.); id10765@alunos.uminho.pt (D.S.A.); pg49123@alunos.uminho.pt (J.D.D.); susanamonteiro@med.uminho.pt (S.M.); nunosilva@med.uminho.pt (N.A.S.); luisapinto@med.uminho.pt (L.P.); 2 ICVS/3B’s-PT Government Associate Laboratory, 4710-057 Braga/Guimarães, Portugal; 3 IPCA-EST-2Ai, Applied Artificial Intelligence Laboratory, Polytechnic Institute of Cávado and Ave, Campus of IPCA, 4750-810 Barcelos, Portugal

**Keywords:** astrocyte, tridimensional reconstruction, immunofluorescence labeling, antibody penetration

## Abstract

Astrocytes are ubiquitous in the brain and spinal cord and display a complex morphology important for the local interactions with neighboring cells, resulting in the modulation of circuit function. Thus, studies focusing on astrocyte physiology in the healthy and diseased brain generally present analyses of astrocytic structure. The labeling method used to visualize the astrocytic structure defines the morphological level to observe and may vary depending on the anatomical sub-regions. The method choice may significantly affect our understanding of their structural diversity. The main goal of this work was to identify a straightforward and efficient protocol for labeling and reconstructing a detailed astrocytic structure to apply and validate in different brain tissue preparations across laboratories. For that, we explored different tissue processing protocols before GFAP labeling to determine the most effective method for reconstructing astrocytic backbones in the mouse hippocampus. Our results show that the reconstruction of astrocytic structure in vibratome sections labeled by free-floating immunofluorescence protocol provides a more practical method to achieve a higher level of detail and arbor complexity in astrocyte backbone reconstruction. Free-floating immunofluorescence labeling is the most reliable method for obtaining better antibody penetration and more detailed astrocyte structure. Finally, we also show that introducing an antigen retrieval step appears useful for visualizing more complete structural details.

## 1. Introduction

Astrocytes are ubiquitous in the brain and spinal cord and are crucial for circuits function and behavior [1,2,3,4]. These cells display a complex morphology ranging from multiple main processes stemming from the soma to endfeet and a myriad of leaflets that are in close contact with synapses and other cellular structures [5,6,7]. Astrocyte morphology displays regional heterogeneity that is essential for the local interactions with neurons and other cells or tissues [8,9,10]. Moreover, the shape, density, and connectivity of astrocytes can change significantly in response to disease or injury [11,12,13,14,15], making the study of astrocyte morphology essential to understanding their roles in brain function.

This field is focused on uncovering these variations, emphasizing the need for accurate methods to assess the different levels of astrocyte structure [16]. The labeling method defines morphological level to observe and may vary depending on the anatomical sub-regions [17]. This issue is particularly critical for brain tissue that does not express genetically encoded reporters, where cells must be labeled by immunohistochemistry (IHC). Therefore, the techniques used to visualize astrocytes can significantly affect our understanding of their diversity, with different methods sometimes yielding different results even in the same brain region.

Several methods are currently available for analyzing and quantifying the complex structure of astrocytes, with the choice of tool depending on the specific research question, available resources, and brain region of interest [18,19]. Detailed three-dimensional analysis of astrocytes often requires sophisticated software and hardware, but many of these tools are expensive and time-consuming. Recently, our group has validated the Simple Neurite Tracer (SNT), a semi-automated, free tool that efficiently quantifies key features of astrocyte backbone such as process length, number of processes, and branching complexity through Sholl analysis [20]. SNT has been successfully applied in various studies, confirming its effectiveness and reliability [10,21,22,23,24]. 

For astrocyte visualization, we utilized the glial fibrillary acidic protein (GFAP) to label the astrocyte backbone for 3D reconstruction. Anti-GFAP immunolabeling, which highlights the main and medium-size branches and partially the cell body, is a popular method for examining astrocyte morphology in physiological and disease conditions [22,25]. However, the results and efficiency depend strongly on the specific experimental conditions used to process the tissue samples and label GFAP.

We evaluated the impact of the choice of slicing method between cryostat and vibratome on the level of astrocytic reconstruction. Cryostat is used to section frozen tissue at lower temperatures, normally requiring tissue embedding. It allows the possibility of producing thinner sections and provides cellular and molecular stability for particularly challenging IHC protocols. On the other hand, vibratome allows slicing in an aqueous solution, and it avoids fixation and embedding steps, which accelerates the processing protocol, but it is not recommended at a thickness lower than 30 μm [26,27]. In both cases, the sections can be processed all the way as free-floating sections, or they can be attached to a slide before the histological procedure. However, to process them in a slide, floating sections must be dried onto the slides, which can produce tissue damage. That is why vibratome slices are typically processed as free-floating sections, and only before the observation are they placed onto slides, dehydrated, and coverslipped using a mounting medium.

Our study explored different tissue processing techniques before GFAP labeling to determine the most effective method for reconstructing astrocytic backbones in the mouse hippocampus. Our goal was to identify a straightforward and efficient protocol for labeling and reconstructing a detailed astrocytic structure, simplifying the study of their morphology, and applying and validating in different tissue preparations across laboratories.

## 2. Materials and Methods

### 2.1. Animals

All procedures involving mice were performed according to the guidelines for the welfare of laboratory mice as described in Directive 2010/63/EU. In addition, they were approved by the Local Ethics Committee (ORBEA 004/2018) and the National Authority for Animal Experimentation, DGAV (DGAV 023838). Mice were group-housed in standard cages (3 to 6 mice per cage) with food and water ad libitum. The housing room was at 22 °C with controlled ventilation and under a light/dark cycle of 12 h (lights on from 8 AM to 8 PM). To assess the morphology of astrocytes in the dorsal hippocampus, coronal brain slices were obtained from C57BL/6J mice (*n* = 4). Thy1-GFP mice [28] were used to assess the fluorescent intensity of the immunofluorescence labeling in different preparations. These mice express EGFP under the control of neuron-specific elements from the Thy1 gene for detailed characterization. Thy1 is an immunoglobulin superfamily member expressed by projection neurons in many parts of the nervous system, which is helpful in this study as it highly and steadily expresses EGFP throughout the depth of the brain slice.

### 2.2. Tissue Processing

Brain samples were processed as previously described [29,30,31]. C57BL/6J and Thy1-GFP mice were deeply anesthetized with a mixture of ketamine (75 mg/kg) and medetomidine (0.5 mg/kg), followed by intracardiac perfusion with saline and 4% paraformaldehyde (PFA). Brains were carefully removed, immersed in 4% PFA for 36 h at room temperature (RT), and then transferred to a 30% sucrose solution at 4 °C until sinking. 

Sectioning with the cryostat: brains were then cryopreserved in Neg-50 Frozen Section medium (ThermoFisher Scientific, Waltham, MA, USA) at −20 °C until sectioning (thickness: 20 μm or 30 μm; Leica CM1900, Wetzlar, Germany). Part of the brain slices were processed in slides, thawed, and dried at RT for 10 min before the immunolabeling protocol, while the remaining brain slices were processed as free-floating. 

Sectioning with the vibratome: brains were immersed in a 3% agarose solution and then sectioned (thickness: 30 μm; Leica VT1000S, Wetzlar, Germany). Brain slices were maintained in phosphate-buffered saline (PBS) until the free-floating immunolabeling protocol. 

IHC protocol: brain slices were washed in PBS solution followed by permeabilization with 0.3% Triton-X100 (Sigma Aldrich, St. Louis, MO, USA) in PBS solution (0.3% PBS-T) for 10 min. After permeabilization, the slices were washed in PBS and incubated with the blocking solution of normal goat serum (NGS; 10%) in PBS for one hour at RT. This blocking step was followed by overnight incubation, at 4 °C, with the primary antibody rabbit polyclonal anti-GFAP (1:200; Dako, Glostrup, Denmark) in a 0.3% PBS-T solution with 2% NGS. On the next day, the slices were washed in PBS and incubated with the secondary antibody Alexa Fluor^®^ 488 goat anti-rabbit (1:1000; Invitrogen, ThermoFisher Scientific, Waltham, MA, USA) in a 0.3% PBS-T solution with 2% NGS, for 1 h at RT. After incubation, the slices were rinsed with PBS, and the nuclei were labeled with 4′,6-diamidino-2-phenylindole (DAPI) (1:1000, Invitrogen, St. Bend, OR, USA) for 10 min at RT. Finally, slices were washed with PBS and mounted using Immumount (ThermoFisher Scientific, Waltham, MA, USA). All procedures during this day were performed in the dark.

Antigen retrieval: after permeabilization, part of the 20 μm thick slices sectioned in the cryostat were submitted to an antigen retrieval (AR) step with citrate buffer (10 mM, pH 6.0, Sigma-Aldrich, St. Louis, MO, USA) to test the antigen retrieval procedure. Briefly, slices were placed in a preheated citrate solution (pH 6.0, Sigma Aldrich), incubated twice for 10 min at 100 W microwave potency, and let to cool for 15 min. After the AR step, slides followed the same IHC protocol as described before, including the overnight incubation, at 4 °C, with the primary antibody rabbit polyclonal anti-GFAP (1:200; Dako, Denmark) in a 0.3% PBS-T solution with 2% NGS. Cryosections were stored for one week before performing antigen retrieval.

Brain slices were imaged on an Olympus LPS FV1000 confocal microscope (Olympus, Germany) using the 20× (N.A. 0.70; 1.5 μm of z-step; 1024 × 1024 pixels) and the 60× objectives (N.A. 1.42; 1.0 μm of z-step; 640 × 640 pixels). The images from the CA1 region of the hippocampus were obtained following the coordinates of the mouse brain atlas [32]: dorsal hippocampus, 1.8 ± 0.1 mm posterior to bregma. The brain tissue for each experiment was processed (sectioned, stored, and stained) simultaneously to minimize labeling heterogeneity between samples. Slight labeling differences might be found between figures, considered independent experiments.

### 2.3. Morphological Analysis 

The tridimensional (3D) reconstruction of the astrocytic structure was performed in the following subfields of the hippocampus: CA1 (stratum radiatum and/or stratum lacunosum moleculare). The GFAP-stained astrocytic structure was skeletonized in confocal z-stacks obtained from C57BL/6J. The semi-automatic tool, SNT, an updated version of the Simple Neurite Tracer, a free Fiji-ImageJ software plugin (Version 1.54f 64-bit http://fiji.sc/Fiji), was used. Our laboratory previously validated this tool to quantify different features of astrocytic structure: total process length, number of processes, the complexity of the astrocytic arbor given by Sholl analysis, and the last process intersection given by the maximum intersection radius in the Sholl analysis, as a fiduciary marker to outline limits of the cell [20]. Briefly, the reconstruction of the selected astrocytes began by marking the center of the DAPI-stained nucleus, where every primary process will originate. Next, a distant point within the primary process was selected, and SNT automatically determined the process midline and tortuosity. This step was repeated after confirming the suggested path until the primary process, respective branches, and branchlets were reconstructed entirely. This process was repeated for every primary process. The reconstruction was performed in 1–3 sections per slide, in 2–3 slides per condition.

### 2.4. Fluorescence Intensity Analysis

The intensity of fluorescence signal for both GFAP and EGFP was individually calculated in the DG stratum moleculare using the ImageJ software (Version 1.54f 64-bit http://fiji.sc/Fiji): analyze > set measurements > check both mean grey value and integrated density > ok > ctrl + M (the measurements were calculated for each z positions).

The mean intensity was normalized for the maximum intensity values for each slice, and then plotted against the percentage of the total z-stack acquired.

The stratum DG stratum moleculare was selected over the stratum radiatum due to the uneven distribution of pyramidal cell fibers in the latter, which could potentially skew the intensity analysis.

### 2.5. Statistical Analysis

Statistical analysis was performed using the GraphPad Prism version 8 (GraphPad Software, La Jolla, CA, USA). Parametric tests were applied throughout all datasets, including non-continuous measures, and passed the Kolmogorov–Smirnov test normality test for normal distribution.

Analysis of astrocytic total process length, number of processes, and number of intersections at long distances from the nucleus using different methodologies was performed by Student’s *t*-test or one-way ANOVA. A two-way ANOVA and Tukey’s multiple comparison tests were applied for Sholl analysis. The values are presented as mean ± standard error of the mean (SEM), and significant statistical differences were considered when *p* ≤ 0.05.

## 3. Results

### 3.1. GFAP Immunolabeling in Vibratome Sections Displays a Higher Level of Detail for Astrocyte Reconstruction

**Figure 1:**
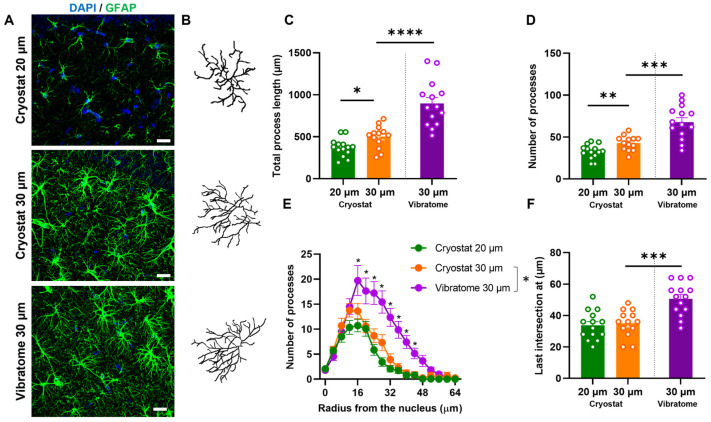
GFAP immunolabeling in vibratome sections displays a higher level of detail for astrocyte reconstruction. Maximum projection of confocal image z-stacks of GFAP-immunolabeled astrocytes (green) in the CA1 stratum radiatum in cryostat 20 µm (**top**), cryostat 30 µm (**middle**), and vibratome 30 µm (**bottom**) (**A**). Representative 3D reconstruction of astrocytes from cryostat 20 µm, cryostat 30 µm, and vibratome 30 µm (**B**); total process length (**C**), number of processes (**D**), Sholl analysis (**E**), and last intersection radius (**F**). Data plotted as individual astrocyte values (dots) and mean ± SEM (columns and bars). * *p* ≤ 0.05; ** *p* ≤ 0.01; *** *p* ≤ 0.001; **** *p* ≤ 0.0001.

A crucial factor that impacts the reconstruction of the astrocytic backbone is the thickness of the slice. Previous work from our group and others performed analysis on 20 µm thick cryosections. To evaluate the impact of slice thickness on the reconstruction of the astrocytic backbone, we opted to increase the slice thickness to 30 µm, followed the slide IHC protocol outlined in the methods section, and then compared the reconstructed astrocytes to those from a 20 µm thick slice (Figure 1A,B). The analysis revealed that, as expected, by increasing the thickness of the tissue to 30 µm, the reconstruction of the astrocytic backbone is more detailed. In particular, the astrocytes reconstructed in 30 µm thick slices present higher total process length (*p* = 0.0100, t = 2.786) and number of processes (*p* = 0.0053, t = 3.055) than astrocytes from tissue sectioned at 20 μm of thickness (Figure 1C,D). Additionally, the Sholl analysis confirmed that astrocytes in both conditions display a typical tree-like structure with increasing intersections until 12–16 μm distance from the nucleus, followed by a steady decrease (Interactionthickness–radius: F_16,425_: 1.088, *p* = 0.3643, η2: 0.01; Radius: F_16,425_: 49.55, *p* < 0.0001, η2: 0.63; Thickness: F_1,425:_ 15.89, *p* < 0.0001, η2: 0.01) (Figure 1E). Two-way ANOVA post-hoc analysis showed a difference in arbor complexity between the two conditions only at 28 µm from the soma (*p* = 0.0383). Finally, no significant differences were observed in the distance from the soma to the final process intersection between conditions (Figure 1F). This implies that increasing the tissue thickness to 30 μm does not enhance the detection of territory size details. Thus, by increasing the thickness of the tissue, we slightly increase the level of detail of the astrocyte reconstruction in terms of total process length and number of processes but not territory size.

These findings demonstrate the suitability of cryostat sectioning for later astrocyte reconstruction from brain tissue. However, cryostat sectioning requires tissue frizzing, which can affect the quality and integrity of the tissue and is time-consuming. To circumvent these disadvantages, we tested a quicker and less intrusive method: vibratome sectioning followed by free-floating immunofluorescence labeling. We reconstructed astrocytes from 30 μm vibratome sections and compared them with reconstructions from cryosections described above. This analysis revealed that astrocytic backbones from tissue sectioned with the vibratome were more complex than astrocytes from tissue sectioned in the cryostat (Figure 1A,B). Specifically, we observed a significant increase in astrocytic total process length (*p* < 0.0001, t = 4.682—Figure 1C) and the number of processes (*p* = 0.0003, t = 4.181—Figure 1D). As expected, Sholl analysis showed that astrocytes from both groups present an increase in intersections until 16 μm distance from the soma, followed by a steady decrease in complexity (Interactionsectioning method–radius: F_16,425_: 3.940, *p* < 0.0001, η2: 0.05; Radius: F_16,425_: 35.32, *p* < 0.0001, η2: 0.50; Sectioning: F_1,425_: 58.15, *p* < 0.0001, η2: 0.05). Interestingly, two-way ANOVA posthoc analysis revealed that astrocytes reconstructed in vibratome sections were more complex than those from cryosections from 16 to 40 μm from the soma (*p* ≤ 0.01, Figure 1E). Ultimately, the distance from the soma to the last process intersection was greater in astrocytes reconstructed from tissue sectioned with the vibratome than those from the cryostat (*p* = 0.0003, t = 4.197—Figure 1F).

In summary, the reconstruction of astrocytic structure in vibratome sections followed by free-floating immunofluorescence protocol provides a higher level of detail and arbor complexity in astrocyte backbone reconstruction.

### 3.2. Free-Floating GFAP Immunolabeling Increases the Level of Detail for Astrocyte Reconstruction

**Figure 2:**
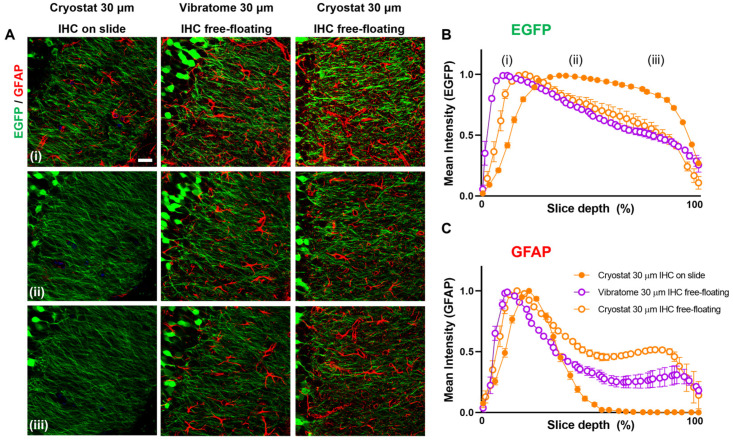
Free-floating GFAP IHC increases the level of detail in astrocyte reconstruction. (**A**) Confocal images of Thy1-EGFP mice at different z-stack depths of the stratum DG moleculare, top (i) to bottom (iii) in the 3 conditions: cryosections followed by the immunolabelling procedure on glass slides or free-floating, and vibratome sections followed by free-floating immunolabelling. Mean EGFP (**B**) and GFAP (**C**) fluorescent intensity distribution across different slice depths in the three conditions: cryostat 30 μm IHC on slide (full orange dot), cryostat 30 μm IHC free-floating (empty orange dot), and vibratome 30 μm IHC free-floating (empty purple dot).

To elucidate the reasons behind the heightened level of detail and complexity in tissue reconstruction when using the vibratome compared to the cryostat, we conducted a fluorescent intensity analysis on hippocampal slices obtained from Thy1-EGFP mice, where EGFP is constitutively expressed in Thy1+ neurons, being stable along the slice depth. For comparison, we performed GFAP IHC on both cryostat and vibratome slices, as described in Figure 1. This allowed us to compare the fluorescence intensity of genetically encoded EGFP, which is unaffected by the IHC protocol, with that of GFAP, which directly reflects the efficiency of the IHC protocol (see Figure 2A). Here, we compared 3 conditions: cryosections followed by the immunolabelling procedure on glass slides or free-floating, and vibratome sections followed by free-floating immunolabelling. Due to variations in the number of z-stack images between conditions, the mean fluorescence intensity was plotted against the percentage of slice depth per image.

The graph in Figure 2B depicts the distribution of EGFP intensity across the slice depth, from top (i) to bottom (iii). As we can observe, the mean EGFP intensity distribution is similar between the three conditions, displaying the same fluorescence distribution between the IHC performed free-floating after cryostat sectioning or after vibratome sectioning. In both conditions, the EGFP signal intensity starts higher at the upper surface of the slice (i), followed by a gradual decline (ii) until the bottom of the slice (iii). In the cryosections processed by immunolabeling on slide, the EGFP signal also starts higher at the surface of the slice (i), in this case displaying a slower decrease to the bottom.

On the other hand, as expected, the distribution of GFAP fluorescence intensity differs significantly between the free-floating and on-slide IHC conditions (see Figure 2C). In the conditions using free-floating IHC after either cryostat or vibratome sectioning, GFAP fluorescence intensity peaks at the slice surface, with a slower decrease until the middle of the slice (ii) and remaining stable at 30–50% intensity until the end of the z-stack. Strikingly, in cryosections processed by IHC on the slide, the GFAP signal peaks at the slice surface, sharply declining to zero at around 50% of the total depth. It is noteworthy that the labeling of cryosections resulted in higher intensities at lower depths, suggesting that the sectioning method and pre-sectioning process of the tissue might also influence IHC results.

These findings suggest that the main factor affecting the fluorescence intensity distribution across the depth of the slice is performing the GFAP IHC free-floating rather than on the slide, regardless of the sectioning method. This may be due to enhanced antibody penetration in both slice surfaces when free-floating as compared to the one-sided contact on the slide.

### 3.3. Tridimensional Reconstruction of Less Complex Astrocyte Backbones in Cryosections Still Allows for Identifying Significant Structural Changes

**Figure 3:**
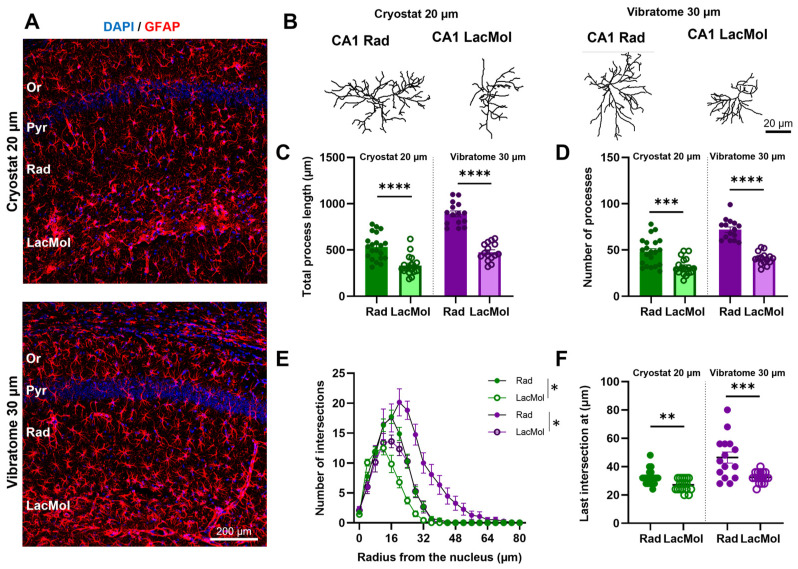
Tridimensional reconstruction of less complex astrocyte backbones in cryosections still allows for identifying significant structural changes. Maximum projection of confocal image z-stacks of GFAP-immunolabeled astrocytes (red) in the CA1 stratum radiatum and lacunosum moleculare in cryostat 20 µm (**top**) and vibratome 30 µm (**bottom**) (**A**). Representative 3D reconstruction of astrocytes from cryostat 20 µm and vibratome 30 µm of both CA1 layers (**B**); characterization of astrocyte 3D structures by analysis of total process length (**C**), number of processes (**D**), Sholl analysis (**E**), and last intersection radius (**F**). In dark green astrocytes reconstructed from CA1 stratum radiatum in cryostat 20 µm, in light green astrocytes reconstructed from CA1 lacunosum moleculare in cryostat 20 µm, in dark purple astrocytes reconstructed from CA1 stratum radiatum in vibratome 30 µm, and in light purple astrocytes reconstructed from CA1 lacunosum moleculare in vibratome 30 µm. Data plotted as individual astrocyte values (dots) and mean ± SEM (columns and bars). * *p* ≤ 0.05; ** *p* ≤ 0.01; *** *p* ≤ 0.001; **** *p* ≤ 0.0001.

As described above, the reconstruction of astrocytic backbones in vibratome slices labeled using free-floating immunofluorescence yields more detailed tridimensional measures. Indeed, our laboratory recently used this procedure to show that astrocytes display structural heterogeneity across the hippocampal subfields of CA1 and DG [10]. This study found that astrocytes from the CA1 radiatum and DG moleculare display more complex backbones than astrocytes from CA1 Or, CA1 lacunosum moleculare, and DG Hil. Since many laboratories in the field must work with cryopreserved tissue, namely when working with models of disease or tissue obtained from banks, we questioned whether structural changes could still be observed in thinner cryosections. We conducted GFAP IHC on hippocampal cryosections (20 µm thick) to address this question. We then compared the reconstructed astrocytes from layers CA1 radiatum and CA1 lacunosum moleculare of the mouse hippocampus with those reconstructed using 30 µm thick vibratome slices (see Figure 3A–F).

As we can observe in Figure 3C, sectioning the tissue with cryostat at 20 µm is still reliable in outlining significant differences between CA1 layers astrocytes.

In line with this, in the tissue processed with cryostat at 20 µm, CA1 radiatum astrocytes presented more processes (*p* = 0.0003, t = 4.042) and total process length (*p* < 0.0001, t = 4.980) than CA1 lacunosum moleculare astrocytes (Figure 3C,D). The Sholl analysis confirmed that in both cryostat (20 µm) and vibratome (30 µm), astrocytes in the layer CA1 radiatum and lacunosum moleculare display a typical tree-like structure with an increasing number of intersections until 12–16 μm distance from the nucleus, followed by a steady decrease (Statistics for 20 μm thick cryosections: Interactionlayer–radius: F_20,801_: 12.69, *p* < 0.0001, η2: 0.05; Radius: F_20,801_: 170.9, *p* < 0.0001, η2: 0.73; Layer: F_1,801_: 77.84, *p* < 0.0001, η2: 0.01. Statistics for 30 μm thick vibratome sections: Interactionlayer–radius: F_20,588_: 4.273, *p* < 0.0001, η2: 0.04; Radius: F_20,588_: 55.83, *p* < 0.0001, η2: 0.6; Layer: F_1,588_: 92.90, *p* < 0.0001, η2: 0.05) (Figure 3E). Two-way ANOVA post-hoc analysis validated the greater complexity of astrocyte arbors in CA1 radiatum compared to CA1 lacunosum moleculare between 12 and 28 µm from the soma in 20 µm thick cryosections (*p* ≤ 0.01) and between 20 and 40 µm from the soma in 30 µm thick vibratome slices (*p* ≤ 0.01).

Ultimately, in both conditions, the distance from the soma to the last process intersection was greater in astrocytes reconstructed from CA1 radiatum than those from the CA1 lacunosum moleculare (p20 µm = 0.0011, t20 µm = 3.528; p30 µm = 0.0009, t30 µm = 3.669—Figure 1F).

These findings confirm that despite the decrease in complexity obtained, the tridimensional reconstructions using immunofluorescence labeling of 20 µm thick cryosections can capture significant differences in astrocyte morphology, including total process length, number of processes, and arbor complexity, when comparing with reconstructions in sections obtained through the same histological procedure.

### 3.4. Antigen Retrieval in 20 µm Cryosections Enhances the Detail of Astrocytic Backbone Reconstructions

**Figure 4:**
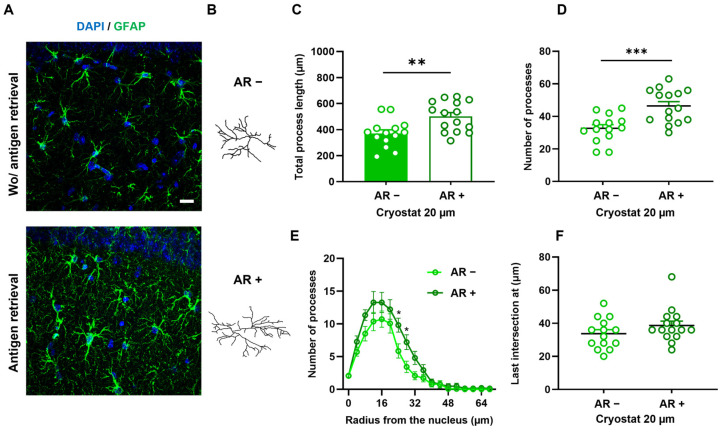
Antigen retrieval in 20 µm cryosections enhances the detail of astrocytic backbone reconstructions. Maximum projection of confocal image z-stacks of GFAP-immunolabeled astrocytes (green) in the CA1 stratum radiatum in cryostat 20 µm without (**top**) and with (**bottom**) antigen retrieval (**A**). representative 3D reconstruction of astrocytes from cryostat 20 µm without (**top**) and with (**bottom**) antigen retrieval (**B**); characterization of astrocyte 3D structures by analysis of total process length (**C**), number of processes (**D**), Sholl analysis (**E**), and last intersection radius (**F**). Data plotted as individual astrocyte values (dots) and mean ± SEM (columns and bars). * *p* ≤ 0.05; ** *p* ≤ 0.01; *** *p* ≤ 0.001.

We have validated the reliability of processing tissue with a cryostat at a thickness of 20 µm to be still able to detect structural differences. However, the decrease in the level of detail in the reconstruction observed might hinder the detection of structural changes when the differences are less striking. This led us to investigate how we could enhance reconstruction detail when the sole option is to utilize cryopreserved tissue. We then tested including an antigen retrieval (AR) step in the IHC protocol. This option has been demonstrated to heighten immunolabeling specificity in tissue [33]. Consequently, we compared astrocytic structures reconstructed in layer CA1 stratum radiatum of the hippocampus from cryosectioned slices at 20 µm, labeled with and without an antigen retrieval step (see Figure 4A–F).

The antigen retrieval was performed as previously described [20]. Briefly, slices were placed in a preheated citrate solution (pH 6.0, Sigma Aldrich), incubated twice for 10 min at 100 W microwave potency, and let to cool for 15 min. As we can observe in Figure 4A,B, employing antigen retrieval increases the level of detail of astrocytic backbone reconstruction. In detail, performing antigen retrieval increases the total process length (*p* = 0.0035, t = 3.201) and number of processes (*p* = 0.0005, t = 3.924) of astrocytes reconstructed (Figure 4C,D). The Sholl analysis (Figure 4E) confirmed that both conditions (presence or absence of AR) display a typical tree-like structure with increasing intersections until 12–16 μm distance from the nucleus, followed by a steady decrease (Interactionantigen retrieval–radius: F_17,486_: 1.441, *p* = 0.1125, η2: 0.01; Radius: F_17,486_: 59.92, *p* < 0.0001, η2: 0.65; Antigen retrieval: F_1,486_: 25.99, *p* < 0.0001, η2: 0.01). Two-way ANOVA posthoc analysis revealed that performing antigen retrieval increases the level of complexity of the astrocytic arbor detected, particularly at 24 and 28 µm distances from the soma (*p* = 0.0146) (Figure 4F). Interestingly, the analysis of astrocytic backbones in tissue processed by the drastic antigen retrieval yielded structural parameters similar to those obtained by the more straightforward processing of 30 µm thick vibratome slices (Figure 1) in all parameters analyzed. This suggests that the antigen retrieval step is a valid alternative to obtain sufficient detail in the morphological reconstruction of these cells for laboratories that must work with cryopreserved tissue.

## 4. Discussion

This study explores various tissue processing methods before GFAP immunofluorescent labeling to optimize the reconstruction of astrocyte backbones while minimizing potential tissue damage and time consumed in the task. We emphasize GFAP immunolabeling, allowing the intricate reconstruction of astrocytic backbone complexity [20]. Despite the availability of alternative techniques, GFAP immunolabeling remains the most widely utilized method for screening astrocytic structure changes due to its cost-effectiveness and efficiency in time management [10,29,34].

Our findings reveal that a simple adjustment, such as increasing tissue thickness from 20 to 30 µm, yields a more comprehensive reconstruction of astrocytic structures. The decision regarding tissue thickness depends mainly upon the purpose of the analysis and the available tools. Notably, rodent hippocampal astrocyte territories typically span diameters of approximately 40 to 60 μm [7,10,35], indicating that at both 20 and 30 μm, a large extent of the astrocytic structure is lost. However, if time and tissue availability constraints are not a concern, extending tissue thickness at least to 100–200 μm and employing clearing techniques to enhance antibody penetration in such thick slices could be advantageous [36]. Furthermore, we did not consider animal models that express a fluorescence reporter under astrocytic promoters, even if their slice thickness can be increased without needing antibody penetration. This decision aligns with our aim to establish an easily adaptable, rapid, and reproducible protocol applicable across various animal models without generating new transgenic models.

Subsequently, we sought to optimize time-consuming steps by slicing the brain tissue using the vibratome and performing the immunofluorescence protocol in free-floating conditions. This protocol allowed faster and less demanding slicing, compared to the cryostat, without the need to freeze the tissue. Our findings revealed that astrocyte reconstructions performed in vibratome-sectioned tissue exhibited greater complexity across all assessed parameters—total process length, number of processes, and distance from the soma to the last intersection. Notably, Sholl analysis demonstrated a notable increase in arbor complexity between 16 and 40 μm from the soma. In summary, this protocol offers enhanced detail and complexity in astrocytic backbone reconstruction compared to the cryostat method while being less harmful to the tissue and time-consuming [37].

We conducted a fluorescence intensity analysis along the Z-axis to investigate the underlying reasons for the difference in backbone complexity obtained in 30 μm brain slices from the cryostat and vibratome and the different IHC processing. This analysis unveiled the expected differential antibody penetration levels between the two preparations, with notably more efficient penetration observed in sections processed free-floating. This disparity is expected, as the free-floating method allows antibodies to penetrate sections from all angles. This should also lead to reduced background staining and better signaling during imaging due to more effective washing. The decision to perform the IHC on glass slides is usually needed when dealing with thin slices, as the free-floating method is not recommended for sections less than 30 μm, as they risk fragmenting within the well [27]. It is important to note that using vibratome or cryostat also depends on the type of tissue analyzed. For instance, brain slices composed of different sub-regions that can easily detach, such as those containing the ventral hippocampus or the choroid plexus, require extra caution during vibratome slicing to prevent detachment from the rest of the slice [37]. In conclusion, increasing tissue thickness enhances reconstruction detail, but the transition to free-floating IHC is required for optimal results. Moreover, quantifying the fluorescence intensity along the Z-axis is an excellent method to control the GFAP labeling across experimental conditions effectively.

Subsequently, we observed that sectioning the tissue with cryostat at 20 µm is still reliable in outlining significant differences between CA1 layers astrocytes. This discovery is precious for researchers who must work with 20 μm cryosections, as it suggests that this process still detects changes in astrocyte structure between conditions [38]. Furthermore, our findings indicate that when working exclusively with 20 µm thick cryosections, implementing antigen retrieval enhances the level of detail in astrocytic backbone reconstruction to similar levels of free-floating processing. PFA fixation induces covalent cross-linking among molecules and cellular structures, thereby consolidating them into a non-soluble network, preserving cells or their constituents. It maintains the chemical and physical properties of the cells and allows antibodies to penetrate intracellular structures, being a valuable preparation for immunolabeling protocols [39]. In the conditions used in our work, antigen retrieval influenced the unmasking of GFAP epitopes, significantly enhancing the fluorescence signal in fixed tissue. Consequently, researchers facing limitations in tissue availability and restricted to cryopreserved materials can benefit from an antigen retrieval procedure to augment reliability and fluorescence intensity. However, it is crucial to note that this drastic method can be harsh for the tissue, potentially compromising protein structure and tissue integrity and, consequently, the accuracy of structural measurements obtained. Moreover, microwave heating can be difficult to maintain; therefore, its application should be carefully considered and fully controlled.

While GFAP excels in identifying the primary processes of most astrocytes in various brain regions, it is crucial to acknowledge its significant limitations. One notable limitation is the absence of a reliable marker that can consistently label all astrocytes across different brain regions. GFAP does not extend throughout the entire astrocyte, lacking presence in the distal and intricately branched arbor processes, astrocyte nuclei, and most cell somas [19]. The researcher should consider another labeling method if the goal is to reconstruct the entire astrocytic arbor complexity until astrocytic leaflets (which is beyond the scope of this article).

In conclusion, reconstructing astrocytic structure in vibratome sections followed by a free-floating immunofluorescence protocol provides straightforward processing, rendering a higher level of detail and arbor complexity in astrocyte backbone reconstruction. Free-floating immunofluorescence labeling is the most reliable method for obtaining better antibody penetration and more detailed astrocyte structure. In case researchers must work with thinner cryopreserved sections, the antigen retrieval option appears useful for visualizing more complete structural details.

## Data Availability

The data presented in this study are available upon request from the corresponding author.
